# Strontium- and calcium-containing, titanium-stabilised phosphate-based glasses with prolonged degradation for orthopaedic tissue engineering

**DOI:** 10.1177/0885328215588898

**Published:** 2015-09

**Authors:** Mustafa Al Qaysi, Nick J Walters, Farzad Foroutan, Gareth J Owens, Hae-Won Kim, Rishma Shah, Jonathan C Knowles

**Affiliations:** 1 Division of Biomaterials and Tissue Engineering, UCL Eastman Dental Institute, London, UK; 2 Department of Electronics and Communication Engineering, Tampere University of Technology, Tampere, Finland; 3 Adult Stem Cell Group, Institute of Biomedical Technology, University of Tampere, Tampere, Finland; 4 BioMediTech (Institute of Biosciences and Medical Technology), Tampere, Finland; 5 Department of Chemistry, Faculty of Mathematical and Physical Sciences, University College London, London, UK; 6 Department of Nanobiomedical Science and BK21 Plus NBM Global Research Center for Regenerative Medicine, Dankook University, Cheonan, Republic of Korea; 7 Institute of Tissue Regeneration Engineering and College of Dentistry, Dankook University, Cheonan, Republic of Korea; 8 Unit of Orthodontics, Department of Craniofacial Growth and Development, UCL Eastman Dental Institute, London, UK

**Keywords:** Phosphate-based glass, strontium, calcium, biomaterial, tissue engineering

## Abstract

Strontium- and calcium-releasing, titanium-stabilised phosphate-based glasses with a controlled degradation rate are currently under development for orthopaedic tissue engineering applications. Ca and/or Sr were incorporated at varying concentrations in quaternary phosphate-based glasses, in order to promote osteoinduction. Ti was incorporated at a fixed concentration in order to prolong degradation. Glasses of the general formula (P_2_O_5_)–(Na_2_O)–(TiO_2_)–(CaO)–(SrO) were prepared via the melt-quench technique. The materials were characterised by energy-dispersive X-ray spectroscopy, X-ray diffraction, ^31^P magic angle spinning nuclear magnetic resonance, Fourier transform infrared spectroscopy, differential thermal analysis and density determination. The dissolution rate in distilled water was determined by measuring mass loss, ion release and pH change over a two-week period. In addition, the cytocompatibility and alkaline phosphatase activity of an osteoblast-like cell line cultured on the surface of glass discs was assessed. The glasses were shown to be amorphous and contained Q^1^, Q^2^ and Q^3^ species. Fourier transform infrared spectroscopy revealed small changes in the glass structure as Ca was substituted with Sr and differential thermal analysis confirmed a decrease in crystallisation temperature with increasing Sr content. Degradation and ion release studies also showed that mass loss was positively correlated with Sr content. These results were attributed to the lower electronegativity of Sr in comparison to Ca favouring the formation of phosphate-based mineral phases. All compositions supported cell proliferation and survival and induced at least 2.3-fold alkaline phosphatase activity relative to the control. Glass containing 17.5 mol% Sr had 3.6-fold greater alkaline phosphatase activity than the control. The gradual release of Ca and Sr supported osteoinduction, indicating their potential suitability in orthopaedic tissue engineering applications.

## Introduction

Osteoporosis is a progressive bone disease, most common in post-menopausal women, in which the delicate balance of bone turnover is disrupted. The porous microarchitecture of osteoporotic bone is caused by simultaneous down-regulation of bone mineral deposition by osteoblasts and up-regulation of mineral resorption by osteoclasts. This results in high susceptibility to bone fractures and is a major cause of morbidity and mortality.^1^

The cost of orthopaedic surgery is a significant and increasing financial burden, with osteoporosis-related fracture alone accounting for estimated direct costs of EUR ∼37 bn in the EU in 2010^2^ and USD ∼17 bn in the US in 2005.^1,3^ These figures are predicted to increase by ∼25 and ∼50%, respectively, by 2025, due to ageing and increasingly diverse population demographics. There is subsequently a high demand for novel biomaterials which induce bone formation for the surgical treatment of fractures and for other orthopaedic and dental tissue engineering applications. A wide range of orthopaedic materials have been developed over the past 50 years for the treatment of fractures caused by osteoporosis, congenital disorders such as osteogenesis imperfecta and cleft palate, or injury. One such class of materials is phosphate-based glasses, which are advantageous over silica-based glasses due to their more controllable dissolution rate.^2,4^ While silica-based glasses typically take several years to dissolve,^5^ the dissolution rate of phosphate-based glasses can be varied over several orders of magnitude, from ∼2 h to >1 year, via the addition of oxide modifiers, such as TiO_2_, which increase the stability of network crosslinks.^6^ This in turn enables precise tuning of the degradation rate for controlled release of ions that promote or inhibit specific cellular process,^7^ e.g. induction of bone formation (Ca^2+^, Sr^2+^, polyphosphates)^6,8,9^; promotion of vascularisation and angiogenesis (Co^2+^ and Cu^2+^)^10,11^ and antimicrobial action (Ag^+^, Cu^2+^, F^−^, Zn^2+^).^12,13^ Furthermore, the tuneable degradation rate of phosphate-based glasses can be targeted to match that of tissue turnover, e.g. bone resorption and deposition, for promotion of osseointegration. Combined with their similar composition to the mineral phase of bone, this makes phosphate-based glasses suitable for various orthopaedic tissue engineering applications, including coatings for dental, maxillofacial and orthopaedic implants and scaffolds for bone tissue engineering.

Ca and Sr are widely known to influence osteoinduction and osteogenesis.^14^ The use of Sr in biomaterials has gained particular interest over the last decade, due to its apparent dual anabolic and anti-catabolic mechanisms of action.^15^ Through these synergistic processes, Sr simultaneously upregulates mineral deposition by osteoblasts and inhibits bone resorption by osteoclasts, resulting in a net gain in mineralised tissue. The oral drug strontium ranelate has been notably successful in reducing hip and vertebral fractures in post-menopausal women by improving bone mineral density.^1,16–19^ Although recommendations regarding its use have recently changed, due to a small but significant increased risk of adverse cardiovascular disorders in patients with cardiovascular contraindications, data do not indicate any detrimental effects in patients without such conditions.^20^ Given that this process appears to be induced by Sr^2+^ cations, the controlled delivery of Sr^2+^ in bone tissue engineering applications is therefore considered to have potentially therapeutic effects.^7,14,21–23^

The effect of strontium content has previously been investigated in silica-based melt-quench glasses. Increasing Sr content in glass discs with the formula (SiO_2_)_46.5_–(P_2_O_5_)_1_–(Na_2_O)_26.4_–(CaO)_(23.1-x)_–(SrO)_x_ (x = 0, 2.3, 11.5 or 23.1) (mol%) resulted in increased cell proliferation and normalised alkaline phosphatase (ALP) activity in human osteosarcoma cell line Saos-2. Sr content also inhibited calcium phosphate resorption and reduced tartrate-resistant acid phosphatase activity in a dose-dependent manner, suggesting a dual action mechanism of released Sr^2+^ ions.^24^ Recently, an in vivo study demonstrated the effectiveness of a Sr-containing bioactive glass with the formula (CaO)_3.18_–(SiO_2_)_51.45_–(MgO)_8.67_–(Na_2_O)_4.62_–(K_2_O)_4.62_–(ZnO)_3.47_–(P_2_O_5_)_5.20_ (mol%) in inducing bone formation in rabbits.^23^

Furthermore, in a series of studies, our group developed complex ternary, quaternary and quinternary phosphate-based glasses containing 0, 1, 3 and 5 mol% Sr and 0, 3 and 5% Ti.^6,8,9^ Increasing Ti content resulted in a direct increase in glass stability and cytocompatibility, as expected. More complex trends, however, were observed in the case of Sr. A threshold of 1 mol% appeared to disrupt the glass network and cause substantially more rapid degradation than was observed in Sr-free compositions. This reduced the viability of cells grown on the surface of the materials. Increasing Sr content to 3 or 5 mol%, however, resulted in a lesser increase in degradation and an increase in cytocompatibility.^6,9^ These trends were attributed to disruption of the glass network caused by the larger atomic radius of Sr^2+^ than Ca^2+^ (effective radii of 1.18 and 1.00 Å, respectively).^6^

In the present study, quaternary and quinternary phosphate-based glasses with Ti fixed at 5 mol% and substantially higher Sr content (up to 35 mol%, compared with 5 mol% in previous studies) were prepared via the melt-quench technique, in order to further investigate the effects of Sr content. The novel glasses had the formula (P_2_O_5_)_50_–(Na_2_O)_10_–(TiO_2_)_5_–(CaO)_(35-x)_–(SrO)_(x)_ (x = 0, 3.5, 17.5 or 35) (mol%). These compositions had 0, 10, 50 and 100 mol% CaO:SrO substitution. The physical properties of each glass composition were characterised using energy-dispersive X-ray spectroscopy (EDX), X-ray diffraction (XRD), ^31^P magic angle spinning nuclear magnetic resonance (^31^P MAS-NMR), Fourier transform infrared spectroscopy (FTIR), differential thermal analysis (DTA) and density determination. The degradation of the materials was determined by measuring weight loss, ion release and pH change upon storage in distilled water over a period of two weeks. Cytocompatibility and ALP activity of osteoblast-like cell line MG-63 were also assessed over the same period.

## Materials and methods

### Materials

The following chemical precursors were used without further purification: diphosphorus pentoxide (P_2_O_5_, 98%, VWR, Lutterworth, UK), sodium dihydrogen phosphate (NaH_2_PO_4_, 99%, VWR), titanium oxide (TiO_2_, 99%, VWR), calcium carbonate (CaCO_3_, 98.5%, VWR) and strontium carbonate (SrCO_3_, 98.5%, BDH Laboratory Supplies, Poole, UK).

### Glass preparation

The precursors were mixed thoroughly (Stomacher 400 Circulator, Seward, Worthing, UK) according to the four compositions summarised in Table 1. The mixtures were placed in a 200 ml Pt/10%Rh crucible (type 71040, Johnson Matthey, Royston, UK) and heated to 1350℃ for 4 h (RHF 1500, Carbolite, Hope, UK), after which the melted glass was poured into a preheated graphite cylindrical mould (ø 15 mm) and heated at 420℃ for 1 h. The glass was then left to gradually cool to room temperature overnight, in order to remove residual stress. Glass rods were then released from the mould and cut into 2 mm thick discs (Accutom 50 electric diamond saw, Struers, Catcliffe, UK). The glass was ground to form a powder for XRD, ^31^P MAS-NMR and FTIR analyses (MM 301 Mixer Mill, Retsch GmbH, Hope, UK).

**Table 1.** Theoretical glass compositions.

Glass code	Full theoretical composition	Concentration (mol%)				
		P_2_O_5_	Na_2_O	TiO_2_	CaO	SrO
Sr0	P_50_Na_10_Ti_5_Ca_35_	50	10	5	35	0
Sr3.5	P_50_Na_10_Ti_5_Ca_31.5_Sr_3.5_	50	10	5	31.5	3.5
Sr17.5	P_50_Na_10_Ti_5_Ca_17.5_Sr_17.5_	50	10	5	17.5	17.5
Sr35	P_50_Na_10_Ti_5_Sr_35_	50	10	5	0	35

### Materials characterisation

#### EDX

EDX (Inca 300, Oxford Instruments, Abingdon, UK) was used to determine the exact compositions of the prepared samples. SEM (XL30, Philips, Eindhoven, Netherlands) was operated at 20 kV, spot size 5 and a working distance of 10 mm, in order to identify the particular elements and their relative proportions with EDX from the scanned area.

#### Density determination

The density of each composition was measured using an analytical balance and density determination apparatus according to Archimedes’ principle (AG 204 & MS-DNY-43, Mettler Toledo, Beaumont Leys, UK). Ethanol (99.8%, Sigma-Aldrich, Gillingham, UK) was used as the immersion vehicle, since phosphate-based glasses are water soluble. The density of the glass discs (n = 3) was calculated using equation (1)

(1) $$\rho_{\mathrm{glass}}=\frac{m_{\mathrm{air}}}{{\text{m}}_{\text{air}}-{\text{m}}_{\text{ethonal}}}\times \rho_{\mathrm{ethonal}}$$

where *ρ_glass_*, *ρ_ethanol_*, m_air_ and m_ethanol_ are the density of the specimen (g/cm^3^), density of ethanol at the temperature at which the measurement was performed, mass (g) of the specimen in air and mass of the specimen under ethanol, respectively.

#### XRD

The crystallinity of each glass composition was determined by XRD (D8 Advance Diffractometer, Brüker, Coventry, UK). Specimens of glass powder were positioned in a flat plate geometry and Ni-filtered Cu Kα radiation was used. Data were collected using a Lynx Eye detector with a step size of 0.02° over an angular range of 2θ = 10–100° and a count time of 12 s.

#### ^31^P MAS-NMR

The network structure of each glass composition was determined by ^31^P MAS-NMR (VNMRS-400 Spectrometer, Varian, Crawley, UK). Spectra of each glass composition were recorded at 161.87 MHz and referenced to the resonance of the secondary reference ammonium dihydrogen phosphate (NH_4_H_2_PO_4_) at 0.9 ppm (relative to 85% H_3_PO_4_ solution at 0 ppm). Specimens of glass powder were loaded into a 4 mm (rotor o.d.) magic angle spinning probe. Between 20 and 88 spectra were obtained for each specimen using direct excitation with a 90° pulse and 60 s recycle delay at ambient probe temperature (∼25℃) and at a sample spin rate of 10 kHz. Spectra were processed using DM-fit software.

#### FTIR

The infrared band assignment of each glass composition was determined by FTIR (FT-IR 2000 and Timebase software, Perkin Elmer, Seer Green, UK). Specimens of glass powder were placed on an attenuated total reflectance accessory (Golden Gate, Specac, Orpington, UK) and spectra in the range of 2000–600 cm^−1^ were acquired at room temperature in absorbance mode.

#### DTA

The thermal properties of each glass composition were determined by DTA (Labsys, Setaram, Caluire, France). The study was carried out on 50 mg of each glass composition from 300℃ up to 1200℃ at a heating rate of 20℃/min, in order to determine the glass transition (T_g_), crystallisation (T_c_) and melting (T_m_) temperatures. Blank run was also carried out to baseline correct the data.

### Material degradation

The degradation of the glass discs (n = 3) was quantified after one, four, seven and 14 days storage in 25 ml deionised water (pH 7.0 ± 0.1) by measuring the mass change of the specimens, the ion release and the pH change of the solution. At each time point, specimen extracts were collected for pH change (Orion Star pH Meter, Thermo Scientific, UK) and ion chromatography measurements. Glass samples were dried and weighed at each time point for determination of mass change, before being transferred to fresh deionised water. Mass change was calculated according to equation (2)

(2) $$\Delta \text{m}=\frac{{\text{m}}_{0}-{\text{m}}_{\text{t}}}{A}$$

where Δm, m_0_, m_t_ and A are the change in mass (g), initial mass, mass at specific time point and surface area of the specimen (cm^2^), respectively.

#### Ion chromatography

Distilled water containing glass dissolution products were collected at each time point and the release of cations (Na^+^, Ca^2+^ and Sr^2+^) and anions (${\text{PO}}_{4}^{3-}$, ${\text{P}}_{2}{\text{O}}_{7}^{4-}$, ${\text{P}}_{3}{\text{O}}_{9}^{3-}$ and ${\text{P}}_{3}{\text{O}}_{10}^{5-}$) was quantified using ion chromatography, as previously described.^25^ Prior to cation analysis, liquid specimens were passed through OnGaurd IIA filters (Dionex, Thermo Scientific, Hemel Hempstead, UK) and elution was performed within 20 mM methanesulfonic acid (Sigma-Aldrich). Cations and anions were analysed under suppressed conductivity (ICS-1000 system equipped with CS12A column and ICS-2500 system equipped with AS16 column, respectively, Dionex). Isocratic separation was applied for analysis of anions with a linear KOH gradient of 30–50 mM over a 35 min period (EG50 eluent generator) and data were extrapolated from standard curves (Chromelon software, Dionex), prepared using commercial standard solutions (Dionex) and results expressed as cumulative release per unit area of the individual specimen tested (cm^2^).

### Cell studies

#### Cell culture

Human osteoblast-like osteosarcoma cell line (MG-63, European Collection of Cell Cultures, Porton Down, UK) was cultured under standard conditions (37℃, 95% air, 5% CO_2_, 95% relative humidity) in Dulbecco’s modified Eagle medium (Gibco, Life Technologies, Paisley, UK) supplemented with 10% foetal bovine serum (Gibco) and 1% penicillin/streptomycin (PAA Laboratories, GE Healthcare, Chalfont St. Giles, UK).

#### Cytocompatibility assay

Cytocompatibility was assessed in accordance with ISO 10993-5:2009.^26^ Glass discs were sterilised under ultraviolet light for 10 min on each side, before being transferred to 48 well plates. MG-63 cells were seeded on top of the discs at a density of 30,000 cells/cm^2^ in 1 ml medium. The control consisted of cells seeded directly on tissue culture plastic (TCP) of the same surface area. Cell culture medium was replenished every three days. Cytocompatibility was assessed after one, four, seven and 14 days. At each time point, cells were seeded at various densities, 2 h prior to the assay, for preparation of standard curves. The medium was then aspirated from the samples and standards and replaced with 1 ml medium containing 10 vol% water soluble tetrazolium salt-8 (WST-8, Cell Counting Kit-8, Sigma Aldrich, Gillingham, UK). WST-8 is a pink substrate which is metabolised by mitochondria to form an orange formazan product. After 80 min incubation at 37℃, absorbance at 460 nm (with a reference wavelength of 650 nm) was measured (Infinite M200, Tecan, Männedorf, Switzerland). Apparent cell density of the samples was then extrapolated from the standard curves.

#### ALP activity

The ALP activity of cells cultured on glass discs for four, seven or 14 days was analysed using a *p*-nitrophenyl phosphate-based assay (SensoLyte *p*NPP Alkaline Phosphatase Assay Kit, AnaSpec, Seraing, Belgium). Medium was aspirated from end-point samples from the cytocompatibility study and the cells were gently rinsed twice using 300 µl assay buffer. The cells were then lysed by freeze–thawing (two cycles of −80℃ for 20 min, followed by 37℃ for 12 min) in 300 µl assay buffer containing 0.2 vol% Triton X-100. After agitation at 4℃ for 10 min, the contents of the cells were collected by repeatedly rinsing the surface using the lysate and centrifuging (2500 × g, 10 min, 4℃). The supplied ALP standard was diluted to various concentrations for the preparation of a standard curve. Fifty microlitres of each supernatant or standard was added to 50 µl *p*NPP substrate in a 96-well plate. After 30 min incubation at 37℃, absorbance was measured at 405 nm. ALP concentration was extrapolated from the standard curve and normalised to the cell densities obtained using the WST-8 assay.

## Results

### Material properties

#### EDX

The measured compositions of prepared glasses by EDX are reported in Table 2. The results showed a reduction of around 3 mol% P_2_O_5_ content compared to the theoretical values. This reduction was accompanied by a concomitant increase in the percentage content of the other oxides to compensate.

**Table 2.** Measured compositions of prepared glasses, determined by EDX.

Glass code	Concentration (mol%)				
	P_2_O_5_	Na_2_O	TiO_2_	CaO	SrO
Sr0	46.4 (±1.1)	10.9 (±0.8)	5.8 (±0.6)	36.9 (±0.9)	0
Sr3.5	46.7 (±1.0)	10.7 (±0.9)	5.7 (±0.7)	32.8 (±0.8)	4.1 (±0.5)
Sr17.5	47.3 (±1.2)	10.8 (±0.7)	5.5 (±0.5)	18.3 (±0.9)	18.1 (±0.6)
Sr35	47.1 (±1.1)	10.8 (±0.7)	5.6 (±0.5)	0	36.5 (±0.6)

**Table 3.** ^31^P MAS-NMR. Peak parameters of the prepared samples.^8,32^

Theoretical composition	Position (ppm, ±0.2)	Q^i^ species	Line width (ppm, ±0.2)	Abundance (%, ±1.0)
P_50_Na_10_Ti_5_Ca_35_	−8.9	1	4.7	0.6
	−25.7	2	12.8	97.0
	−34.2	3	6.7	2.4
P_50_Na_10_Ti_5_Ca_31.5_Sr_3.5_	−8.7	1	4.9	5.8
	−25.6	2	12.6	96.3
	−33.8	3	6.2	1.2
P_50_Na_10_Ti_5_Ca_17.5_Sr_17.5_	−8.4	1	5.2	3.9
	−24.9	2	12.3	95.7
	−33.6	3	5.8	0.4
P_50_Na_10_Ti_5_Sr_35_	−8.3	1	5.6	5.9
	−23.6	2	11.6	94.1
	−8.9	1	4.7	0.6

#### Density

**Figure 1.**
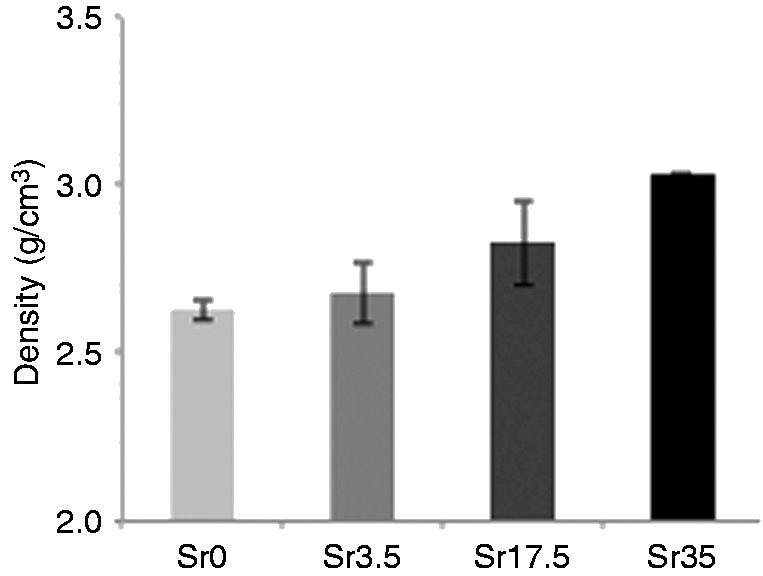
Density of Ti-stabilised phosphate-based glasses with varying Ca and Sr content. Increasing Sr content correlated with increasing density, from 2.6 g/cm^3^ (Sr0) to 3.0 g/cm^3^ (Sr35). Error bars are SD (n = 3).

An increase in Sr content directly correlated with an increase in glass density (Figure 1), with densities ranging from 2.6 g/cm^3^ (Sr0) to 3.0 g/cm^3^ (Sr35).

#### XRD

**Figure 2.**
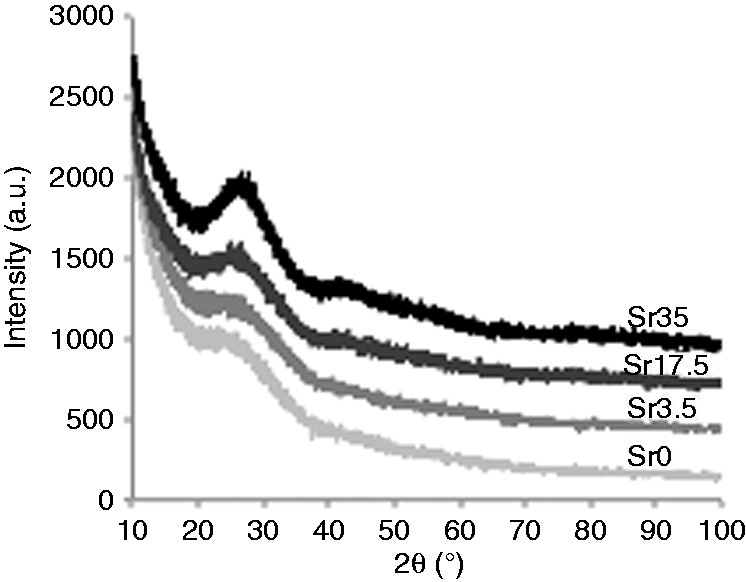
XRD spectra of Ti-stabilised phosphate-based glasses with varying Ca and Sr content. No crystalline phase was detected, indicating the amorphous nature of the glasses.

Figure 2 shows the XRD spectra of the glasses. A broad peak at 2θ values of ∼20–40° confirmed the amorphous nature of all glass samples and indicated that they lacked any detectable crystalline phase.

#### ^31^P MAS-NMR

**Figure 3.**
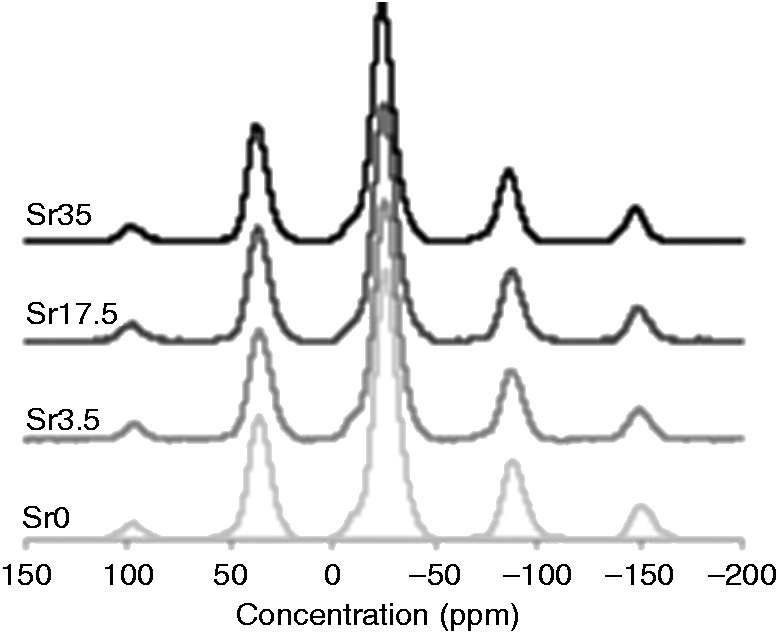
^31^P MAS-NMR spectra of Ti-stabilised phosphate-based glasses with varying Ca and Sr content. Peaks in the range of −8.3 to −8.9 ppm, −23.6 to −25.7 ppm and −33.6 to −34.2 ppm correspond to Q^1^, Q^2^ and Q^3^ phosphate species, respectively.

The ^31^P MAS-NMR spectra of the glass samples are displayed in Figure 3 and the peak parameters are summarised in Table 2. The resonances in ^31^P MAS-NMR spectra are assigned to various Q**^n^** species, where *n* represents the number of bridging oxygen atoms in the ${\text{PO}}_{4}^{3-}$ group that are connected to other such phosphate tetrahedra to form a network.^27^ Peaks in the range of −8.3 to −8.9 ppm are attributed to Q^1^ phosphate units, while peaks in the range of −23.6 to −25.7 ppm and −33.6 to −34.2 ppm correspond to the Q^2^ and Q^3^ species, respectively.^8,28,29^

#### FTIR

**Figure 4.**
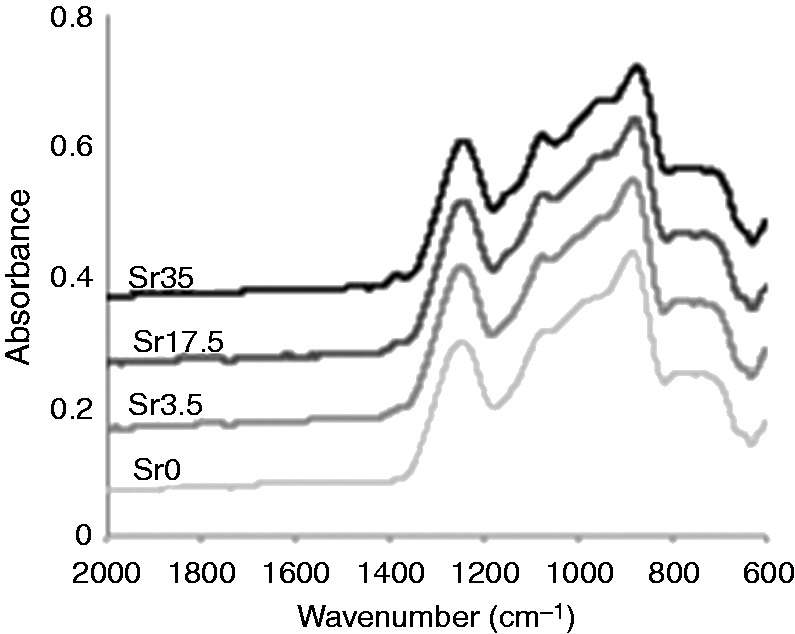
FTIR spectra of Ti-stabilised phosphate-based glasses with varying Ca and Sr content. Broad peaks at 740, 900, 980, 1100 and 1250 cm^−1^ can be assigned to υ_s_ (P–O–P), υ_as_ (P–O–P), υ_s_ (PO_3_)^2−^, υ_as_ (PO_3_)^2−^ and υ_as_ (PO_2_)^−^, respectively.

The FTIR spectra of the phosphate-based glass powders are presented in Figure 4 and their infrared band assignments are summarised in Table 4, according to previous studies on phosphate-based glasses.^28,30,31^ The broad peaks at 740 cm^−1^ can be assigned to symmetrical stretching υs (P–O–P), while the peaks at 900 and 980 cm^−1^ correspond to υas (P–O–P) and υs (PO_3_)^2−^, respectively. The peaks at 1100 and 1250 cm^−1^ can also be assigned to υas (PO_3_)^2−^ and υas (PO_2_)^−^, respectively.

**Table 4.** Infrared band assignment for sol–gel glasses (υ, stretching; _s_, symmetric; _as_, asymmetric).

Wavenumber (cm^−1^)	Assignments	Associated Q^i^
740	υ_s_ (P–O–P)	N/A
900	υ_as_ (P–O–P)	Q^2^
980	υ_s_ (PO_3_)^2−^	Q^1^
1100	υ_as_ (PO_3_)^2−^	Q^1^
1250	υ_as_ (PO_2_)	Q^2^

#### DTA

**Figure 5.**
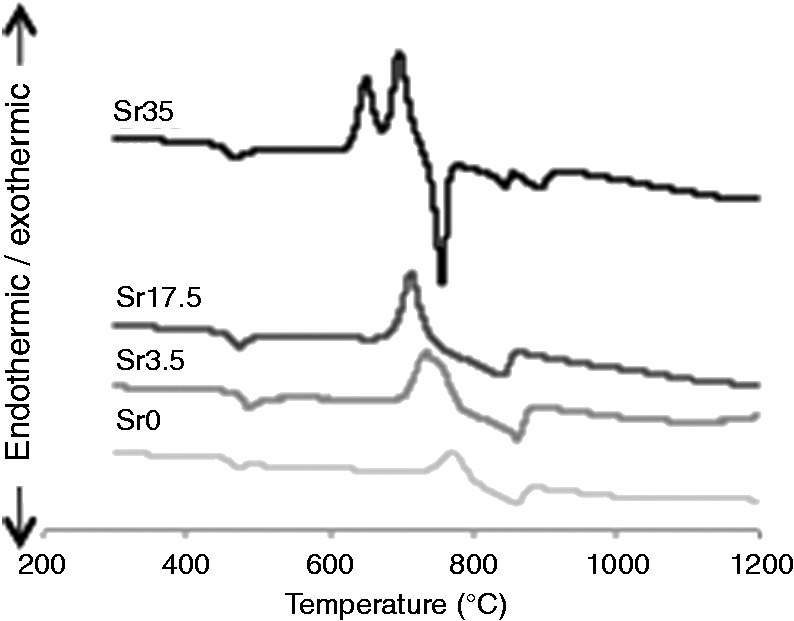
DTA spectra of Ti-stabilised phosphate-based glasses with varying Ca and Sr content. T_g_ was similar (468–483℃) for all compositions. T_c_ declined from a single exothermic peak (761.9℃; Sr0) to two distinct crystallisation peaks (647, 701℃; Sr35). T_m_ similarly declined from a broad peak (850℃; Sr0) to a sharp peak (752℃; Sr35).

Figure 5 shows the DTA spectra of the glasses. The T_g_ of the glasses was in the range of 468–483℃ and there was no clear trend observed as the Sr content was increased. The T_c_, however, declined from a single exothermic peak at 761.9℃ for Sr0 to two distinct crystallisation peaks at T_c_ values of 647 and 701℃ for Sr35. A similar trend was observed for the T_m_ values with decline from a broad peak at 850℃ for Sr0 to a sharp peak at 752℃ for Sr35.

### Glass degradation and ion release

**Figure 6.**
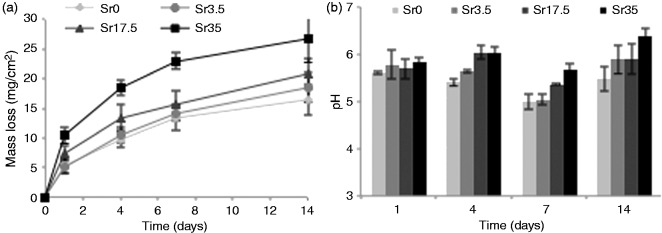
(a) Mass loss and (b) pH change of Ti-stabilised phosphate-based glasses with varying Ca and Sr content, after one, four, seven and 14 days incubation in deionised water. Increasing Sr content caused an increase in mass loss but a lessening reduction of pH. Error bars are SD (n = 3).

The mass loss and pH change of glass specimens is shown in Figure 6. As Sr content was increased, a concomitant increase in degradation was observed, with mass loss after 14 days ranging from 16.4 mg/cm^3^ (Sr0) to 26.7 mg/cm^3^ (Sr35). The difference in mass loss between Sr0 and Sr35 was approximately double that of the difference between Sr0 and Sr17.5, indicating that mass loss was roughly proportional to Sr content. The pH of the storage vehicle decreased continuously over the course of 14 days for Sr0. By increasing Sr content, the effect on pH was lessened, with Sr35 causing minimal reduction in pH.

**Figure 7.**
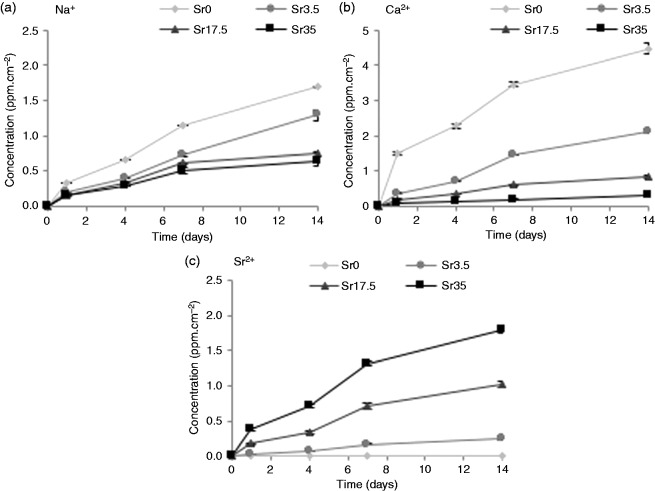
Cumulative release of cations (a) Na^+^, (b) Ca^2+^ and (c) Sr^2+^ from Ti-stabilised phosphate-based glasses with varying Ca and Sr content, after one, four, seven and 14 days incubation in deionised water. Increasing Sr content in the glass resulted in an increase in Sr^2+^ release and a decrease in Na^+^ and Ca^2+^ release. Error bars are SD (n = 3).

**Figure 8.**
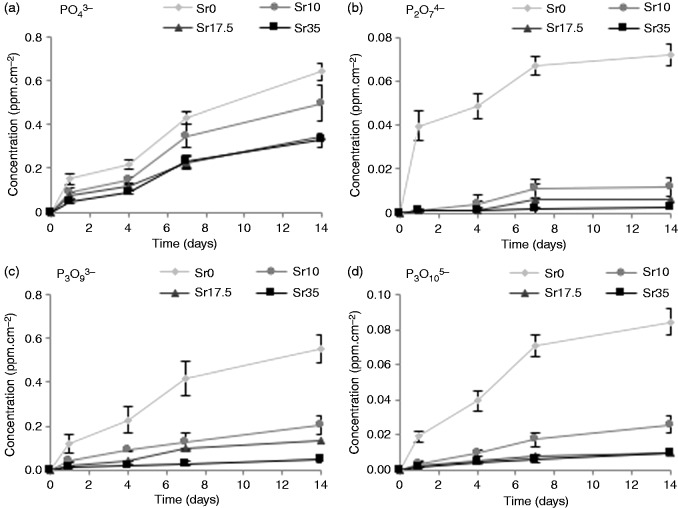
Cumulative release of anions (a) ${\text{PO}}_{4}^{3-}$, (b) ${\text{P}}_{2}{\text{O}}_{7}^{4-}$, (c) , ${\text{P}}_{3}{\text{O}}_{9}^{3-}$ and (d) ${\text{P}}_{3}{\text{O}}_{10}^{5-}$ from Ti-stabilised phosphate-based glasses with varying Ca and Sr content, after one, four, seven and 14 days incubation in deionised water. Increasing Sr content in the glass resulted in a decrease in all polyphosphates. Error bars are SD (n = 3).

The cumulative release of cations (Na^+^, Ca^2+^ and Sr^2+^) and anions (${\text{PO}}_{4}^{3-}$, ${\text{P}}_{2}{\text{O}}_{7}^{4-}$, ${\text{P}}_{3}{\text{O}}_{9}^{3-}$ and ${\text{P}}_{3}{\text{O}}_{10}^{5-}$) is presented in Figures 7 and 8, respectively. The concentration of Na^+^ present in solution decreased as a function of increasing Sr content in the glass. This relationship, however, inversely correlated with weight loss (Figure 6(a)). Sr^2+^ release was found to increase as Sr content in the glass increased. Likewise, Ca^2+^ release correlated directly with Ca content in the glass. A small amount of Ca^2+^ was, however, released from Sr35. Similarly to Na^2+^, polyphosphate ion release was inversely proportional to Sr content. ${\text{PO}}_{4}^{3-}$ appeared to be least affected by Sr content, while ${\text{P}}_{2}{\text{O}}_{7}^{4-}$ exhibited the greatest magnitude in reduction as Sr content was increased. The concentration of ${\text{P}}_{3}{\text{O}}_{9}^{3-}$ and ${\text{P}}_{3}{\text{O}}_{10}^{5-}$ present in solution exhibited a clear reduction as Sr was increased.

### Cytocompatibility and ALP activity

**Figure 9.**
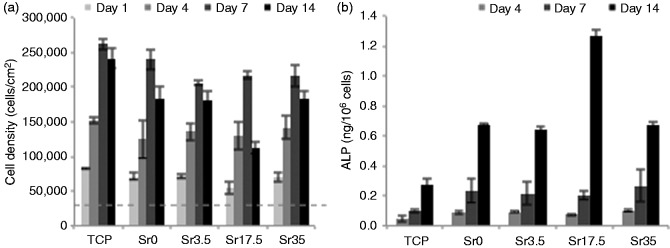
(a) Cell density and (b) normalised ALP activity of MG-63 after culture on Ti-stabilised phosphate-based glass discs with varying Ca and Sr content, for one, four, seven and 14 days. Dotted line represents initial seeding density. TCP is tissue culture plastic control, error bars are SD (n = 3).

All glass compositions had high cytocompatibility (Figure 9(a)). After one and four days, cells cultured on all glass compositions had comparable metabolic activity compared to cells cultured on TCP. Between seeding and day 1, cells approximately doubled in number from 30,000 to ∼70,000 cells/cm^2^. By day 4, they had roughly doubled again to ∼135,000 cells/cm^2^. After seven days, the apparent cell density on Sr0 (240,000 cells/cm^2^) was similar to TCP (262,000 cells/cm^2^) and slightly higher than the other glass compositions (206,000–217,000 cells/cm^2^). By day 14, the density of cells on TCP had dropped slightly, whereas the number of surviving cells on glass compositions fell by between 13 and 48%. The greatest reduction in cell number was observed on Sr17.5.

The ALP activity (Figure 9(b)) of cells cultured on glass discs was higher at all time points than on TCP. ALP activity of cells cultured on glass discs was similar after four and seven days, regardless of composition. After 14 days, however, despite Sr17.5 causing a decrease in overall metabolic activity, the normalised ALP activity was approximately double that of the other compositions.

## Discussion

Research in our group focuses on the development of phosphate-based glasses for tissue engineering applications. Several studies leading up to the present work have investigated the incorporation of Sr in phosphate-based melt-quench glasses. An initial study revealed that tertiary and quaternary glasses with the formula (P_2_O_5_)_50_–(Na_2_O)_(20-x)_–(CaO)_(30)_–(SrO)_(x)_ (x = 0, 1, 3 or 5) (mol%) suffered from poor cytocompatibility due to their rapid degradation rate.^8^ In a follow-up study, incorporation of 3 mol% Ti in quaternary and quinternary (P_2_O_5_)_50_–(Na_2_O)_17_–(TiO_2_)_3_–(CaO)_(30-x)_–(SrO)_(x)_ (x = 0, 1, 3 or 5) (mol%) glasses was shown to prolong the degradation rate and improve cytocompatibility compared to Ti-free compositions.^9^ The substitution of CaO with SrO, however, resulted in an increase in dissolution rate, but with an inverse trend, whereby increasing Sr content had a less pronounced effect, as follows: Sr0 < Sr5 < Sr3 < Sr1. This directly correlated with a decrease in cytocompatibility, with Sr0 best supporting cell growth: Sr0 > Sr5 > Sr3 > Sr1. These unusual but distinctive trends indicated the likelihood of systematic – rather than random – alterations to the glass network. Subsequently, (P_2_O_5_)_50_–(Na_2_O)_15_–(TiO_2_)_5_–(CaO)_(30-x)_–(SrO)_(x)_ (x = 0, 1, 3 or 5) (mol%) compositions, in which Ti content was increased to 5 mol%, were shown to have 2–4 times lower degradation rates than those containing 3 mol% Ti.^6^ The slower dissolution rate also fostered improved cell attachment. Again, degradation was highest in the composition with low Sr content, reaffirming the prominent Sr0 < Sr5 < Sr3 < Sr1 trend observed previously.

More comprehensive analyses of the physical characteristics of glasses with higher Sr and Ti content were performed within the present study. Phosphate-based glasses containing up to 35 mol% Sr were successfully prepared. As previously observed,^8^ glass density was shown to increase as a function of Sr content. This correlates with the significantly higher density of Sr (2.64 g/cm^3^) compared to Ca (1.54 g/cm^3^). The glasses were also shown to be amorphous and to predominantly consist of Q^2^ phosphate units, which slightly decreased as Sr content was increased. In addition, the small peak at around −34 ppm, which corresponds to Q^3^ species, was not present in Sr35. With regards to the FTIR data, as Ca was substituted with Sr, a reduction in the intensity of the peak at 900 cm^−1^ (Q^2^ species) and an increase in the intensity of the peak at 1100 cm^−1^ (Q^1^ species) indicated a move to a more disordered structure. This is likely due to the larger ionic radius of Sr compared to Ca. Furthermore, DTA results confirmed a significant reduction in the T_c_ value as Sr content was increased.

The glass discs were immersed in deionised water for zero, one, four, seven and 14 days and their degradation was analysed by ion chromatography and by measuring the pH change of the water. The use of ion chromatography was favoured over that of inductively coupled plasma spectroscopy, due to its ability to differentiate between different phosphate species. This is a particularly important factor when studying the dissolution of glasses in which phosphate is a major component. Due to the presence of organic solvents which interfere with the ion chromatography column, the use of Tris–HCl was selected against. Phosphate buffered saline, which contains high phosphate concentration, as well as simulated body fluid, which is super-saturated with the same ions as those released from the glasses, was also discounted, as the concentration of released ions would have been indistinguishable from the high background levels.

The detection of a small amount of Ca^2+^ release from Sr35, which should not have contained any Ca, is likely due to impurity of the Sr precursor, which has a purity of 98.5% and contains up to 0.07% Ca. With increasing Sr content, the mass loss at any given time point increased. Overall, there should have been no major change in the glass structure as the Sr^2+^ replaces the similarly charged Ca^2+^. Thus, the overall mass loss may be explained by the difference in the atomic weight between these two divalent cations. The inclusion of a larger ionic radius cation may make the glass network more susceptible to hydrolysis. The Na^+^ release differed in that as Sr^2+^ content was increased, Na^+^ release decreased. The same is seen for PO_4_^3−^, and indeed all the phosphate species measured, and it may be that the Na^+^ and PO_4_^3−^ are reacting once released into solution, making them unavailable for measurement by ion chromatography. Further to this point, the stark reduction in P_2_O_7_^4−^ and P_3_O_10_^5−^ species may be expected, given their high stability constants (i.e. their ability to chelate divalent cationic species).^33^ The assertion that Sr^2+^ release favours mineralisation processes is, to some extent, supported by the pH change data.

All glass formulations were shown to have good cytocompatibility. The apparent cell density after one and four days was similar in all formulations and the TCP control. After seven days, the apparent density of cells surviving on Sr-containing glass discs was ∼20% lower than on TCP or Sr0, but still remained high, at around seven times the original seeding density. By 14 days, the apparent cell density on all samples and TCP had dropped slightly, except Sr17.5, which had halved in number compared to day 7. The ALP activity of cells cultured on glass discs of all compositions was approximately double that of the control at each time point. The one exception to this was Sr17.5, which induced similar ALP activity per cell to other glass formulations at all time points except day 14, by which time it had doubled. This was in spite of the lower apparent cell density on Sr17.5 at 14 days. The decrease in WST-8 metabolism of cells cultured on Sr17.5 is likely caused by the simultaneous increase in ALP activity, rather than an actual reduction in cell density. The higher ALP activity of cells cultured on Sr17.5 fits with previous data concerning the optimal concentration of Sr for induction of osteogenic differentiation of bone marrow stromal cells.^34^ A concentration of 5 µg/ml SrCl_2_ was shown to induce higher levels of COL1A1 expression and calcified matrix deposition than 1 or 10 µg/ml. In the present study, the concentration of Sr^2+^ in extracts of Sr0, Sr3.5, Sr17.5 and Sr35 after 14 days was 0.0, 1.1, 4.5 and 8.1 µg/ml, respectively. The concentration of Sr^2+^ released from Sr35 is therefore higher than optimal and explains the lower ALP activity induced by Sr35 than Sr17.5.

In summary, all glass compositions induced at least 2.3-fold and up to 3.6-fold higher ALP activity than the control at all time points. Furthermore, the cytocompatibility of all formulations was shown to be high. This, combined with their controllable degradation rate, demonstrates the potential of Sr- and Ti-containing phosphate-based glasses for the treatment of osteoporosis, fractures and other orthopaedic, maxillofacial and dental applications.

## Conclusions

Sr- and Ca-containing Ti-stabilised phosphate-based glasses were successfully prepared via the facile melt-quench technique. The materials had an ideal degradation rate for tissue engineering applications, due to the incorporation of Ti, which forms stable crosslinks in the glass network. The glasses also induced between 2.3- and 3.6-fold increase in ALP activity, relative to the control, with Sr17.5 causing the greatest increase, and all compositions had good cytocompatibility. This was likely due to their Ca and Sr content, which are both widely known to support osteoinduction and osteogenesis. In conclusion, Ti-stabilised, Ca- and Sr-releasing phosphate-based glasses have great potential in the treatment of fractures induced by osteoporosis, congenital bone defects and injury, as well as in other orthopaedic, maxillofacial and dental tissue engineering applications.
